# 2,5-Diphenyl-2,5,6,8-tetra­hydro-1,2,4-triazolo[3,4-*c*][1,4]oxazin-4-ium hexa­fluorido­phosphate

**DOI:** 10.1107/S1600536808022599

**Published:** 2008-07-26

**Authors:** Jie Wu, Siping Wei, Bo Liu, Wenhai Wang, Jingbo Lan

**Affiliations:** a Key Laboratory of Green Chemistry and Technology of the Ministry of Education, College of Chemistry, Sichuan University, Chengdu 610064, People’s Republic of China

## Abstract

The title compound, C_17_H_16_N_3_O^+^·PF_6_^−^, is a chiral bicyclic 1,2,4-triazolium salt. In the crystal packing, C—H⋯O and C—H⋯F hydrogen bonds and P—F⋯π contacts [4.078 (11)–4.163 (11) Å, involving the triazolium ring] play an important role in enhancing the stability of the crystal structure.

## Related literature

For related literature, see: Enders & Kallfass (2002 ▶); Fisher *et al.* (2006 ▶); Kerr *et al.*, (2002 ▶); Knight & Leeper (1998 ▶); Readde Alaniz & Rovis, (2005 ▶).
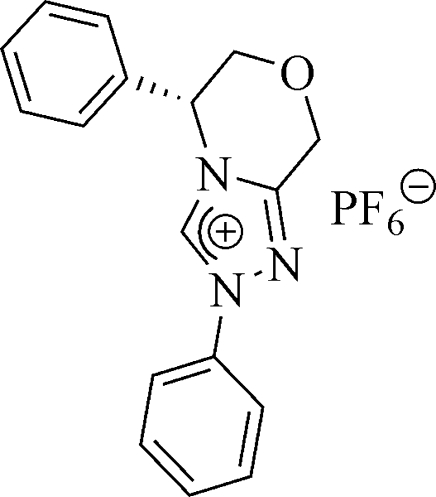


## Experimental

### 

#### Crystal data

C_17_H_16_N_3_O^+^·PF_6_^−^
*M* *_r_* = 423.30
Orthorhombic, 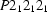

*a* = 8.1706 (6) Å
*b* = 11.4642 (8) Å
*c* = 19.7716 (14) Å
*V* = 1852.0 (2) Å^3^
*Z* = 4
Mo *K*α radiation
μ = 0.22 mm^−1^
*T* = 297 (2) K
0.58 × 0.55 × 0.26 mm

#### Data collection

Bruker SMART CCD area-detector diffractometer
Absorption correction: multi-scan (*SADABS*; Sheldrick, 1996 ▶) *T*_min_ = 0.850, *T*_max_ = 0.961
10481 measured reflections
3632 independent reflections
3040 reflections with *I* > 2σ(*I*)
*R*_int_ = 0.024

#### Refinement

*R*[*F* ^2^ > 2σ(*F* ^2^)] = 0.035
*wR*(*F* ^2^) = 0.096
*S* = 1.20
3632 reflections
257 parameters
H atoms treated by a mixture of independent and constrained refinement
Δρ_max_ = 0.19 e Å^−3^
Δρ_min_ = −0.22 e Å^−3^
Absolute structure: Flack (1983 ▶), 1537 Friedel pairs
Flack parameter: 0.06 (10)

### 

Data collection: *SMART* (Siemens, 1996 ▶); cell refinement: *SAINT* (Siemens, 1996 ▶); data reduction: *SAINT*; program(s) used to solve structure: *SHELXS97* (Sheldrick, 2008 ▶); program(s) used to refine structure: *SHELXL97* (Sheldrick, 2008 ▶); molecular graphics: *ORTEP-3* (Farrugia, 1997 ▶); software used to prepare material for publication: *SHELXTL* (Sheldrick, 2008 ▶).

## Supplementary Material

Crystal structure: contains datablocks global, I. DOI: 10.1107/S1600536808022599/rk2095sup1.cif

Structure factors: contains datablocks I. DOI: 10.1107/S1600536808022599/rk2095Isup2.hkl

Additional supplementary materials:  crystallographic information; 3D view; checkCIF report

## Figures and Tables

**Table 1:** Hydrogen-bond geometry (Å, °)

*D*—H⋯*A*	*D*—H	H⋯*A*	*D*⋯*A*	*D*—H⋯*A*
C3—H3*A*⋯F2^i^	0.98	2.48	3.318 (3)	143
C5—H5*A*⋯O^ii^	0.93 (2)	2.34 (2)	2.899 (3)	118 (2)

Symmetry codes: (i) 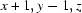; (ii) 
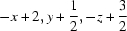
.

**Fig. 1.**
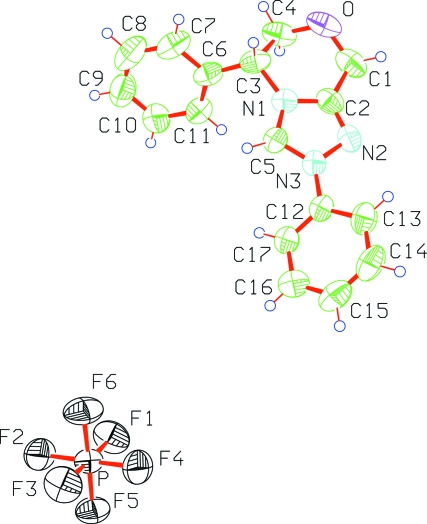
The molecular structure of the title compound with the atom numbering scheme. The displacement ellipsoids are drawn at 50% probability level. The H atoms are presented as a small spheres of arbitrary radius.

## supplementary crystallographic information

### Comment

Recently, triazolium salts which can be used as the precursors of carbenes are widely used in asymmetric catalysis for the C—C bond formation reactions, such as benzoin reactions (Knight & Leeper, 1998; Enders & Kallfass, 2002), Stetter reactions (Kerr *et al.*, 2002; Readde Alaniz & Rovis, 2005) and Diels–Alder reactions (Fisher *et al.*, 2006) owing to of their good stability and excellent catalytic performance. Most researchs illuminate, that chiral bicyclic 1,2,4–triazole carbenes have excellent enantio–selectivity because they have many bulkier groups and show weaker nucleophility than thiazolium and imiazolium salts. The molecular structure of the title compound (Fig. 1) shows that N1—C5—N3 is typical conjugated fragment because both bonds length N1—C5 = 1.330 (3)Å and N3—C5 = 1.322 (3)Å are longer than double bond N2—C2 = 1.296 (3)Å, but shorter than other N—C bonds (1.366 (3)–1.481 (3)Å) . In intermolecular network, P—F···π(*Cg1*) interactions [4.078 (11)–4.163 (11)Å] are the main contributor to the interaction of neighboring layers and play an important part in the connection of the adjacent porous layers in the title crystal structure (*Cg1* is the triazolium centroid). The interatomic C—H···O and C—H···F hydrogen bond are present - see Table.

### Experimental

The title compound was prepared according to the method (Knight & Leeper, 1998; Enders & Kallfass, 2002). A solution of 5–ethoxy–3–phenyl–3,6–dihydro–2*H*–1,4–oxazine (prepared from (*R*)–2–amino–2–phenylethanol) as a colourless liquid was added dropwise to phenylhydrazine hydrochloride (1.44 g, 10 mmol) in methanol (3 ml). The mixture was then stirred for 30 min, followed by addition of triethyl orthoformate (7.4 g, 50 mmol). After being heated at 353 K for 10 h, the reaction mixture was cooled to room temperature and concentrated *in vacuo*. The resulting residue was purified by column chromatography on silica gel with elution with methanol and followed with anion exchange with ammonium hexafluorophosphate to afford the pure triazolium salt as a white solid in 70% yield. Colourless crystals suitable for X–ray analysis were obtained by slow evaporation of acetone solution. ^1^H NMR (400 MHz, DMSO): δ 4.05–4.10 (m, 1H), 4.37–4.41 (m, 1H), 5.22 (dd, *J* = 16 Hz, 16 Hz, 1H), 5.78 (dd, *J* = 6.0 Hz, 6.0 Hz, 1H), 7.47–7.68 (m, 8H), 7.69–7.91 (m, 2H).

### Refinement

All H atoms (except H5*A*) were placed in geometrically idealized positions and constrained to ride on their parent atoms with C—H distances in the range 0.93Å for aryl, 0.97Å for methylene and 0.98Å for methine with U_iso_ = 1.2U_eq_(C).

1537 Friedel pairs were measured.

### Crystal data

**Table:**

C_17_H_16_N_3_O^+^·PF_6_^–^	*F*_000_ = 864
*M**_r_* = 423.30	*D*_x_ = 1.518 Mg m^−^^3^
Orthorhombic, *P*2_1_2_1_2_1_	Mo *K*α radiation λ = 0.71073 Å
Hall symbol: P 2ac 2ab	Cell parameters from 4928 reflections
*a* = 8.1706 (6) Å	θ = 2.5–26.0º
*b* = 11.4642 (8) Å	µ = 0.22 mm^−^^1^
*c* = 19.7716 (14) Å	*T* = 297 (2) K
*V* = 1852.0 (2) Å^3^	Prism, colourless
*Z* = 4	0.58 × 0.55 × 0.26 mm

### Data collection

**Table:**

Bruker SMART CCD area-detector diffractometer	3040 reflections with *I* > 2σ(*I*)
Monochromator: Graphite	*R*_int_ = 0.024
*T* = 297(2) K	θ_max_ = 26.0º
φ and ω scans	θ_min_ = 2.1º
Absorption correction: Multi-scan(SADABS; Sheldrick, 1996)	*h* = −10→7
*T*_min_ = 0.850, *T*_max_ = 0.961	*k* = −14→11
10481 measured reflections	*l* = −24→22
3632 independent reflections	

### Refinement

**Table:**

Refinement on *F*^2^	Hydrogen site location: Geom
Least-squares matrix: Full	H atoms treated by a mixture of independent and constrained refinement
*R*[*F*^2^ > 2σ(*F*^2^)] = 0.035	*w* = 1/[σ^2^(*F*_o_^2^) + (0.05*P*)^2^] where *P* = (*F*_o_^2^ + 2*F*_c_^2^)/3
*wR*(*F*^2^) = 0.096	(Δ/σ)_max_ = 0.001
*S* = 1.20	Δρ_max_ = 0.19 e Å^−^^3^
3632 reflections	Δρ_min_ = −0.22 e Å^−^^3^
257 parameters	Extinction correction: None
Primary atom site location: Direct	Absolute structure: Flack (1983), 1537 Friedel pairs
Secondary atom site location: Difmap	Flack parameter: 0.06 (10)

### Special details

**Table:**

Geometry. All s.u.'s (except the s.u. in the dihedral angle between two l.s. planes) are estimated using the full covariance matrix. The cell s.u.'s are taken into account individually in the estimation of s.u.'s in distances, angles and torsion angles; correlations between s.u.'s in cell parameters are only used when they are defined by crystal symmetry. An approximate (isotropic) treatment of cell s.u.'s is used for estimating s.u.'s involving l.s. planes.
Refinement. Refinement of *F*^2^ against ALL reflections. The weighted *R*–factor *wR* and goodness of fit *S* are based on *F*^2^, conventional *R*–factors *R* are based on *F*, with *F* set to zero for negative *F*^2^. The threshold expression of *F*^2^ > σ(*F*^2^) is used only for calculating *R*–factors(gt) *etc*. and is not relevant to the choice of reflections for refinement. *R*–factors based on *F*^2^ are statistically about twice as large as those based on *F*, and *R*–factors based on ALL data will be even larger.

### Fractional atomic coordinates and isotropic or equivalent isotropic displacement parameters (Å^2^)

**Table:**

	*x*	*y*	*z*	*U*_iso_*/*U*_eq_	
P	0.32742 (6)	0.98825 (5)	0.80826 (3)	0.05508 (16)	
F1	0.2154 (2)	0.87484 (13)	0.80330 (9)	0.0959 (5)	
F2	0.2554 (2)	1.02909 (15)	0.73698 (7)	0.0886 (5)	
F3	0.4387 (2)	1.10126 (14)	0.80938 (10)	0.0966 (5)	
F4	0.4001 (2)	0.94705 (14)	0.87749 (8)	0.0933 (5)	
F5	0.1857 (2)	1.05734 (14)	0.84539 (8)	0.0836 (4)	
F6	0.4689 (2)	0.92073 (17)	0.76896 (9)	0.0978 (5)	
O	0.9022 (2)	−0.21782 (15)	0.73641 (11)	0.0824 (5)	
N1	0.8395 (2)	0.01208 (15)	0.75583 (9)	0.0519 (4)	
N2	0.8073 (2)	−0.00577 (15)	0.86600 (9)	0.0584 (4)	
N3	0.8078 (2)	0.10971 (15)	0.84664 (8)	0.0508 (4)	
C1	0.8303 (4)	−0.19097 (19)	0.79941 (16)	0.0791 (7)	
H1A	0.8925	−0.2275	0.8354	0.095*	
H1B	0.7196	−0.2213	0.8009	0.095*	
C2	0.8273 (3)	−0.06202 (18)	0.80973 (13)	0.0599 (5)	
C3	0.8765 (3)	−0.02665 (18)	0.68603 (12)	0.0617 (6)	
H3A	0.9952	−0.0221	0.6795	0.074*	
C4	0.8266 (3)	−0.1550 (2)	0.68261 (15)	0.0753 (7)	
H4A	0.7085	−0.1616	0.6862	0.090*	
H4B	0.8599	−0.1878	0.6395	0.090*	
C5	0.8292 (3)	0.11963 (19)	0.78062 (10)	0.0510 (5)	
H5A	0.844 (3)	0.188 (2)	0.7561 (11)	0.059 (6)*	
C6	0.7964 (3)	0.04596 (19)	0.63230 (11)	0.0605 (6)	
C7	0.8786 (4)	0.0594 (3)	0.57130 (14)	0.0873 (8)	
H7A	0.9832	0.0287	0.5661	0.105*	
C8	0.8056 (6)	0.1181 (3)	0.51833 (15)	0.1075 (11)	
H8A	0.8602	0.1250	0.4772	0.129*	
C9	0.6547 (5)	0.1658 (3)	0.52585 (14)	0.0951 (10)	
H9A	0.6065	0.2059	0.4902	0.114*	
C10	0.5736 (4)	0.1547 (3)	0.58587 (13)	0.0860 (8)	
H10A	0.4711	0.1887	0.5911	0.103*	
C11	0.6421 (3)	0.0937 (2)	0.63886 (12)	0.0704 (6)	
H11A	0.5843	0.0846	0.6790	0.085*	
C12	0.7902 (3)	0.20118 (18)	0.89483 (9)	0.0519 (5)	
C13	0.8528 (3)	0.1859 (2)	0.95942 (11)	0.0722 (7)	
H13A	0.9029	0.1163	0.9718	0.087*	
C14	0.8388 (4)	0.2766 (3)	1.00491 (13)	0.0846 (8)	
H14A	0.8789	0.2676	1.0486	0.101*	
C15	0.7666 (4)	0.3795 (3)	0.98659 (13)	0.0834 (8)	
H15A	0.7596	0.4407	1.0173	0.100*	
C16	0.7046 (4)	0.3918 (2)	0.92254 (13)	0.0809 (7)	
H16A	0.6546	0.4616	0.9103	0.097*	
C17	0.7151 (3)	0.3032 (2)	0.87617 (11)	0.0645 (6)	
H17A	0.6721	0.3121	0.8329	0.077*	

### Atomic displacement parameters (Å^2^)

**Table:**

	*U*^11^	*U*^22^	*U*^33^	*U*^12^	*U*^13^	*U*^23^
P	0.0415 (3)	0.0612 (3)	0.0625 (3)	0.0005 (2)	−0.0050 (2)	−0.0123 (3)
F1	0.0832 (11)	0.0722 (9)	0.1322 (14)	−0.0225 (8)	−0.0041 (11)	−0.0090 (9)
F2	0.0910 (11)	0.1006 (11)	0.0742 (9)	0.0102 (9)	−0.0220 (8)	−0.0057 (8)
F3	0.0760 (10)	0.0894 (10)	0.1245 (14)	−0.0297 (9)	−0.0093 (10)	−0.0098 (10)
F4	0.0994 (12)	0.1078 (11)	0.0726 (9)	0.0169 (11)	−0.0210 (9)	0.0051 (9)
F5	0.0677 (9)	0.0933 (10)	0.0897 (10)	0.0160 (8)	0.0122 (8)	−0.0164 (8)
F6	0.0666 (9)	0.1201 (13)	0.1066 (12)	0.0267 (10)	0.0073 (8)	−0.0301 (10)
O	0.0625 (10)	0.0646 (10)	0.1201 (15)	0.0171 (9)	−0.0086 (11)	−0.0158 (10)
N1	0.0419 (8)	0.0518 (9)	0.0620 (10)	−0.0072 (9)	0.0024 (7)	−0.0045 (8)
N2	0.0485 (9)	0.0588 (10)	0.0681 (11)	0.0013 (9)	0.0003 (8)	0.0165 (9)
N3	0.0426 (9)	0.0568 (10)	0.0530 (9)	−0.0043 (8)	−0.0002 (8)	0.0044 (8)
C1	0.0693 (15)	0.0540 (12)	0.114 (2)	0.0035 (12)	−0.0008 (17)	0.0067 (14)
C2	0.0417 (10)	0.0570 (11)	0.0810 (15)	0.0025 (10)	−0.0004 (12)	0.0044 (12)
C3	0.0431 (10)	0.0719 (13)	0.0700 (14)	−0.0055 (10)	0.0067 (10)	−0.0197 (12)
C4	0.0557 (13)	0.0661 (13)	0.1042 (19)	0.0062 (12)	−0.0074 (15)	−0.0231 (13)
C5	0.0440 (11)	0.0567 (12)	0.0523 (11)	−0.0105 (10)	0.0021 (9)	0.0015 (9)
C6	0.0557 (13)	0.0633 (13)	0.0627 (12)	−0.0137 (11)	0.0087 (11)	−0.0184 (10)
C7	0.0816 (18)	0.108 (2)	0.0727 (17)	−0.0112 (17)	0.0216 (14)	−0.0218 (16)
C8	0.129 (3)	0.129 (3)	0.0636 (17)	−0.027 (3)	0.025 (2)	−0.0089 (17)
C9	0.126 (3)	0.095 (2)	0.0642 (17)	−0.013 (2)	−0.0022 (18)	0.0077 (14)
C10	0.097 (2)	0.0837 (18)	0.0776 (17)	0.0105 (17)	−0.0036 (16)	0.0053 (14)
C11	0.0725 (16)	0.0786 (15)	0.0602 (13)	−0.0006 (13)	0.0087 (11)	−0.0010 (11)
C12	0.0444 (11)	0.0618 (12)	0.0496 (11)	−0.0096 (10)	0.0010 (8)	0.0024 (9)
C13	0.0699 (16)	0.0886 (17)	0.0582 (13)	−0.0045 (14)	−0.0140 (11)	0.0056 (12)
C14	0.0924 (19)	0.112 (2)	0.0488 (12)	−0.0255 (19)	−0.0122 (13)	−0.0047 (14)
C15	0.092 (2)	0.098 (2)	0.0610 (15)	−0.0213 (18)	0.0092 (13)	−0.0184 (14)
C16	0.091 (2)	0.0764 (16)	0.0754 (15)	0.0059 (16)	0.0039 (15)	−0.0108 (13)
C17	0.0713 (15)	0.0686 (13)	0.0535 (11)	0.0033 (12)	−0.0045 (11)	0.0017 (10)

### Geometric parameters (Å, °)

**Table:**

P—F4	1.5653 (15)	C5—H5A	0.93 (2)
P—F3	1.5829 (17)	C6—C11	1.380 (3)
P—F5	1.5834 (17)	C6—C7	1.389 (3)
P—F1	1.5931 (15)	C7—C8	1.380 (5)
P—F6	1.5935 (18)	C7—H7A	0.9300
P—F2	1.5975 (15)	C8—C9	1.357 (5)
O—C1	1.411 (3)	C8—H8A	0.9300
O—C4	1.425 (3)	C9—C10	1.365 (4)
N1—C5	1.330 (3)	C9—H9A	0.9300
N1—C2	1.366 (3)	C10—C11	1.378 (4)
N1—C3	1.481 (3)	C10—H10A	0.9300
N2—C2	1.296 (3)	C11—H11A	0.9300
N2—N3	1.378 (2)	C12—C17	1.371 (3)
N3—C5	1.322 (3)	C12—C13	1.387 (3)
N3—C12	1.424 (3)	C13—C14	1.379 (4)
C1—C2	1.493 (3)	C13—H13A	0.9300
C1—H1A	0.9700	C14—C15	1.368 (4)
C1—H1B	0.9700	C14—H14A	0.9300
C3—C6	1.500 (3)	C15—C16	1.371 (4)
C3—C4	1.528 (3)	C15—H15A	0.9300
C3—H3A	0.9800	C16—C17	1.371 (3)
C4—H4A	0.9700	C16—H16A	0.9300
C4—H4B	0.9700	C17—H17A	0.9300
			
F4—P—F3	90.95 (10)	C3—C4—H4B	109.7
F4—P—F5	91.32 (9)	H4A—C4—H4B	108.2
F3—P—F5	90.24 (10)	N3—C5—N1	107.02 (19)
F4—P—F1	91.47 (10)	N3—C5—H5A	127.2 (13)
F3—P—F1	177.27 (11)	N1—C5—H5A	125.6 (13)
F5—P—F1	90.95 (9)	C11—C6—C7	118.6 (3)
F4—P—F6	90.26 (10)	C11—C6—C3	123.5 (2)
F3—P—F6	89.29 (10)	C7—C6—C3	117.8 (2)
F5—P—F6	178.36 (10)	C8—C7—C6	120.3 (3)
F1—P—F6	89.45 (10)	C8—C7—H7A	119.9
F4—P—F2	179.08 (10)	C6—C7—H7A	119.9
F3—P—F2	89.10 (10)	C9—C8—C7	120.4 (3)
F5—P—F2	89.60 (9)	C9—C8—H8A	119.8
F1—P—F2	88.46 (10)	C7—C8—H8A	119.8
F6—P—F2	88.83 (9)	C8—C9—C10	119.9 (3)
C1—O—C4	111.6 (2)	C8—C9—H9A	120.0
C5—N1—C2	106.52 (18)	C10—C9—H9A	120.0
C5—N1—C3	129.38 (18)	C9—C10—C11	120.7 (3)
C2—N1—C3	123.73 (18)	C9—C10—H10A	119.6
C2—N2—N3	103.84 (17)	C11—C10—H10A	119.6
C5—N3—N2	110.93 (18)	C10—C11—C6	120.0 (3)
C5—N3—C12	127.64 (18)	C10—C11—H11A	120.0
N2—N3—C12	121.42 (16)	C6—C11—H11A	120.0
O—C1—C2	110.1 (2)	C17—C12—C13	121.4 (2)
O—C1—H1A	109.6	C17—C12—N3	119.56 (18)
C2—C1—H1A	109.6	C13—C12—N3	119.1 (2)
O—C1—H1B	109.6	C14—C13—C12	118.4 (2)
C2—C1—H1B	109.6	C14—C13—H13A	120.8
H1A—C1—H1B	108.2	C12—C13—H13A	120.8
N2—C2—N1	111.67 (18)	C15—C14—C13	120.9 (2)
N2—C2—C1	127.7 (2)	C15—C14—H14A	119.6
N1—C2—C1	120.5 (2)	C13—C14—H14A	119.6
N1—C3—C6	113.87 (18)	C14—C15—C16	119.5 (2)
N1—C3—C4	106.0 (2)	C14—C15—H15A	120.2
C6—C3—C4	112.76 (19)	C16—C15—H15A	120.2
N1—C3—H3A	108.0	C17—C16—C15	121.2 (3)
C6—C3—H3A	108.0	C17—C16—H16A	119.4
C4—C3—H3A	108.0	C15—C16—H16A	119.4
O—C4—C3	109.8 (2)	C16—C17—C12	118.7 (2)
O—C4—H4A	109.7	C16—C17—H17A	120.7
C3—C4—H4A	109.7	C12—C17—H17A	120.7
O—C4—H4B	109.7		

### Hydrogen-bond geometry (Å, °)

**Table:**

*D*—H···*A*	*D*—H	H···*A*	*D*···*A*	*D*—H···*A*
C3—H3A···F2^i^	0.98	2.48	3.318 (3)	143
C5—H5A···O^ii^	0.93 (2)	2.34 (2)	2.899 (3)	118 (2)

Symmetry codes: (i) *x*+1, *y*−1, *z*; (ii) −*x*+2, *y*+1/2, −*z*+3/2.
